# Single‐Cell Transcriptomics Reveals Dynamic Cellular Interactions and Molecular Mechanisms in Myocardial Infarction Recovery

**DOI:** 10.1155/ijog/4888573

**Published:** 2026-03-06

**Authors:** Jianfeng Zhao, Junhui Gong, Cunzhi Zhu

**Affiliations:** ^1^ Department of Cardiology, The People′s Hospital of Danyang (Affiliated Danyang Hospital of Nantong University), Danyang City, China; ^2^ Department of Emergency, Nanjing Tianyinshan Hospital (The First Affiliated Hospital of China Pharmaceutical University), Nanjing City, China

**Keywords:** cell communication, macrophage migration inhibitory factor, myocardial infarction, pseudotime trajectory, single-cell RNA sequencing

## Abstract

**Background:**

Repair and remodeling following myocardial infarction (MI) are complex processes with a wide array of cellular and molecular mechanisms; however, the cell source mediating repair is still poorly understood in terms of heterogeneity and temporal dynamics.

**Methods:**

We performed a single‐cell RNA sequencing (scRNA‐seq) analysis of cardiac tissues from different time points post‐MI, as well as in gene knockout (ChrisKO) and health control groups. The data were mined by UMAP and t‐SNE dimension reduction visualization, pseudotime trajectory analysis, cell communication network analysis, and gene expression pattern cluster.

**Results:**

A collection of cell types contributing to cardiac repair was identified, including fibroblasts, macrophages, endothelial cells, and cardiomyocytes that each expressed gene markers and showed temporal distributions associated with distinct injury phases. Pseudotime trajectory analysis identified a continuous change in cellular state from inflammatory to reparative phase, with immune cells in early stages and tissue repair cells at latter stages. The activation of macrophage migration inhibitory factor (MIF) signaling pathway is highly involved in repair after MI, where chemokine‐secreting cells and cardiac fibroblasts act as major MIF signal sources. Network analysis of the intercellular communication revealed that macrophages are key orchestrators of repair. When analyzing branch‐specific gene expression, we found that several important regulatory factors including Atpdv1h, Lypla1, Mrpl15, Tcea1, Apoa, Cldn1, Dpep1, and Map had changing trends at different phases during regeneration.

**Conclusion:**

Our study profiled a panoramic landscape of cellular and molecular dynamics after MI at single‐cell resolution, demonstrating key cell communication networks and regulatory genes that present novel targets for developing therapeutic strategy toward cardiac repair.

## 1. Introduction

Myocardial infarction (MI) continues to be an important cause of morbidity and mortality globally and poses a serious public health problem despite advances in clinical intervention. The heart has a complex dynamic response to MI, characterized by inflammation, repair, and remodeling of the tissue. The effectiveness of this repair process is an important determinant of long‐term cardiac function and patient survival, as maladaptive remodeling frequently progresses to heart failure with associated increased mortality [1–3].

The cellular geography of the postinfarcted heart is profoundly heterogeneous and temporally evolving. The first of these is an inflammatory phase, where immune cells, particularly neutrophils and macrophages, infiltrate the area to clear necrotic debris and produce cytokines that regulate subsequent repair [4–6]. This phase evolves into a proliferative stage, where fibroblasts and endothelial cells as well as other resident cardiac cells are activated resulting in scar formation with tissue reorganization. Although these general phases have been well defined, the specific cellular heterogeneity, molecular axes, and intercellular signaling networks orchestrating successful cardiac repair are not completely understood. Classical bulk transcriptomic studies have been informative in identifying global gene expression alterations upon MI, but they do not possess the required cellular resolution to highlight contributions from specific cell types and rare cell populations that could be important for repair. The recent development of single‐cell RNA sequencing (scRNA‐seq) has transformed our capacity to dissect complex biological systems at an unprecedented resolution as it allows characterization of individual cell states, developmental trajectories, and intercellular communication networks [7–9].

Recent research has made use of scRNA‐seq to identify and compare cell types between the healthy and diseased heart, illustrating a more diverse cellular landscape than initially anticipated [10–12]. Nevertheless, thorough characterization of the dynamic cellular and molecular changes in MI healing is lacking. Knowledge of these dynamics is important for identification of critical cellular transitions and molecular pathways that could be targeted therapeutically to promote cardiac repair and prevent adverse remodeling. Gene knockout (ChrisKO) affords us the opportunity to ascertain the functional significance of these identified molecular pathways through genetic manipulation and, therefore, define causative associations between specific pathways and repair‐dependent outcomes. The ChrisKO mouse was created by state‐of‐the‐art CRISPR/Cas9 gene editing to knock out a key control gene required for repairing and remodeling damaged hearts. This manipulation allows a systematic study of pathway‐specific influences on the cellular landscape during MI recovery. We performed the MI surgical procedure on ChrisKO mice and their respective wild‐type (WT) counterparts, followed by a 14‐day postinfarction resection of the cardiac tissue for scRNA‐seq. We used scRNA‐seq to characterize the transcriptome of the infarcted heart from ∼7 up to 30 dpi, compared with ChrisKO and healthy controls. Integrating computational methods such as dimensionality‐reduction algorithms, pseudotime trajectory analysis, intercellular communication modeling, and branch‐specific gene expression to systematically analyze the cellular dynamics and molecular mechanisms underlying cardiac repair following MI, we aimed to identify crucial signaling networks (emphasis on a central macrophage migration inhibitory factor [MIF] signaling network) and illustrate the orchestration role of macrophages in directing repair response. Our results establish a valuable high‐resolution cellular state and transition map in the recovery from post‐MI, which can align key regulatory molecules and cell–cell interaction networks guiding this process. They may also contribute to the design of new approaches for specifically targeting therapies to optimize cardiac repair process, which will benefit millions of myocardial infarct patients in the world.

## 2. Materials and Methods

### 2.1. Construction and Sequencing of Single‐Cell RNA Libraries

Single‐cell suspensions were processed using the 10x Genomics Chromium Single Cell 3 ^′^ Gene Expression platform according to the manufacturer′s instructions. Isolated single cells were initially encapsulated into droplets with barcoded beads, followed by cell lysis and reverse transcription to produce barcoded cDNA. After amplification, cDNA libraries were fragmented and end‐repaired with A‐tailing before ligation of adapters. Library sequencing was conducted using the Illumina NovaSeq 6000, and about 50,000 reads per cell were sequenced to guarantee enough sensitivity for gene detection.

### 2.2. scRNA‐seq Data Preprocessing

Raw sequencing reads were analyzed using the Cell Ranger pipeline (10x Genomics) to generate gene count matrices and perform barcode processing, UMI counting, and alignment of sequenced fragments against mouse reference genome mm10. Computation of quality control metrics included genes detected per cell (nFeature_RNA), unique molecular identifiers per cell (nCount_RNA), and mitochondrial gene content percentages (percent.mt). Downstream analysis was performed on cells with extremely low gene counts (< 500 genes), unreasonably high gene counts (> 5000 genes, possible doublets), or a high percentage of mitochondrially encoded reads (> 20%, indicative that the cell is stressed and/or dying). Normalization: Global‐scaling normalization was performed by normalizing the gene expression measurements for each cell by the total expression, based on a scale factor (10,000), and log‐transformation [13, 14].

### 2.3. Dimensionality Reduction and Cell Type Annotation

Downstream analysis of the quality‐filtered data employed the Seurat R package. Identification of highly variable genes occurred based on their expression and dispersion, with principal component analysis (PCA) performed on these genes. Both jackstraw analysis and elbow plot assessment evaluated the statistical significance of principal components, with the first 15 significant PCs retained for subsequent analyses. Two complementary nonlinear dimensionality‐reduction techniques provided visualization capability: t‐distributed stochastic neighbor embedding (t‐SNE) and uniform manifold approximation and projection (UMAP). A graph‐based clustering approach with optimized resolution parameters performed cell clustering. A combination of established lineage‐specific marker genes and differential expression analysis between clusters conducted cell type annotation. Major cell types receiving identification included cardiac fibroblasts, chemokine‐secreting cells, endothelial cells, macrophages, mesenchymal cells, neuronal cells, pancreatic‐like cells, proliferating cells, T cells, and vascular cells. Validation of each cluster′s identity occurred through examining the expression patterns of canonical markers and by comparison with published cardiac single‐cell datasets [15, 16].

### 2.4. Pseudotime Trajectory Analysis

Reconstruction of the developmental trajectories of cells during cardiac repair employed pseudotime analysis using the Monocle3 R package. Transcriptomic similarity forms the basis for this approach ordering cells along a trajectory, enabling the inference of developmental progressions. The highly variable genes identified during dimensionality reduction constructed the trajectory. Principal graph learning identified the branching structure of the cellular differentiation paths. Cells received assignment to developmental states based on their position along the trajectory, with root states determined based on the expression of early response genes and the enrichment of cells from early time points (7 dpi). Identification of branch points occurred as key cell fate decision nodes, with cells assigned to specific branches for downstream branch‐specific analyses [17, 18].

### 2.5. Cell–Cell Communication Network Analysis

The CellChat R package inferred intercellular communication networks, quantifying cell–cell interactions based on the expression of known ligand–receptor pairs. Performance of the analysis occurred both globally across all identified cell types and specifically for selected populations of interest (fibroblasts, endothelial cells, cardiomyocytes, and macrophages). Network diagrams visualized communication patterns where nodes represent cell types and edges represent the strength of predicted interactions. Detailed examination specifically focused on the MIF signaling pathway, with source and target cells identified based on the expression of MIF and its receptors. Heatmaps and network diagrams quantified and visualized communication strength [19, 20].

### 2.6. Gene Expression Analysis Along Pseudotime and Across Branches

Analysis of gene expression dynamics along the pseudotime trajectory identified temporally regulated genes during cardiac repair. Hierarchical clustering organized genes based on their expression patterns across pseudotime, resulting in the identification of distinct gene modules with similar temporal dynamics. Gene Ontology enrichment analysis performed for each module identified associated biological processes. Identification of genes differentially expressed between developmental branches emerged through branch‐specific gene expression analysis. Scatter plots and violin plots visualized expression levels of key regulatory genes (Atpdv1h, Lypla1, Mrpl15, Tcea1, Apoa, Cldn1, Dpep1, Map, and P16) across pseudotime and different branches, with points colored by cell type and different shapes indicating branch membership.

### 2.7. Statistical Analysis

R (Version 4.0.3) performed all statistical analyses. The Wilcoxon rank‐sum test with Bonferroni correction for multiple comparisons assessed differential gene expression between clusters. Genes with an adjusted *p* value < 0.05 and log2 fold change > 0.25 received consideration as significantly differentially expressed. Jackstraw analysis with 100 iterations evaluated the statistical significance of principal components. For trajectory analysis, a likelihood ratio test comparing a full model (including branch information) to a reduced model assessed significance of branch‐specific gene expression. For all analyses, statistical significance was defined as *p* values less than 0.05.

## 3. Results

### 3.1. scRNA‐seq Analysis of MI Reveals Transcriptomic Dynamics

A comprehensive scRNA‐seq investigation examines MI across multiple temporal stages (7, 14, and 30 dpi), incorporating both ChrisKO and healthy control specimens. In Figure 1a, violin plot representations demonstrate how gene features (nFeature_RNA), RNA molecule counts (nCount_RNA), and mitochondrial gene percentages (percent.mt) distribute across experimental groups, revealing that 1000–3000 detected genes characterize the majority of cells. A robust correlation (0.92) between gene features and RNA counts emerges in Figure 1b, where each experimental group displays distinctive distribution patterns—particularly noteworthy are the ChrisKO cells (blue), which manifest unique transcriptomic signatures. The relationship between principal components and standard deviation appears in Figure 1c, establishing that most data variation concentrates within the first 10 PCs. Through gene expression variability analysis in Figure 1d, 2000 highly variable genes (red) contrast with 13,753 nonvariable genes (black), with labeled key regulatory genes encompassing Alas2, Cd2, Csf1r‐Cyfip1, Rps5, Mmp3, and Myb. The transcriptomic landscape during postinfarction cardiac recovery gains valuable illumination through these collective results, which spotlight potential molecular regulators governing myocardial repair mechanisms.

Figure 1Single‐cell RNA sequencing analysis of myocardial infarction reveals transcriptomic dynamics. (a) Data quality assessment and preprocessing. (b) Principal component analysis and gene expression patterns. (c) Cellular heterogeneity and population dynamics. (d) Cellular composition and intercellular communication networks.

**Figure:**
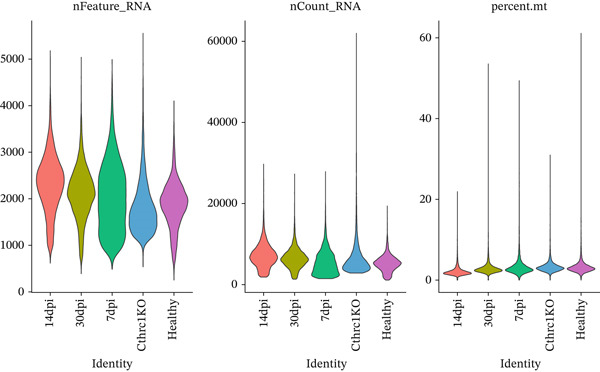
(a)

**Figure:**
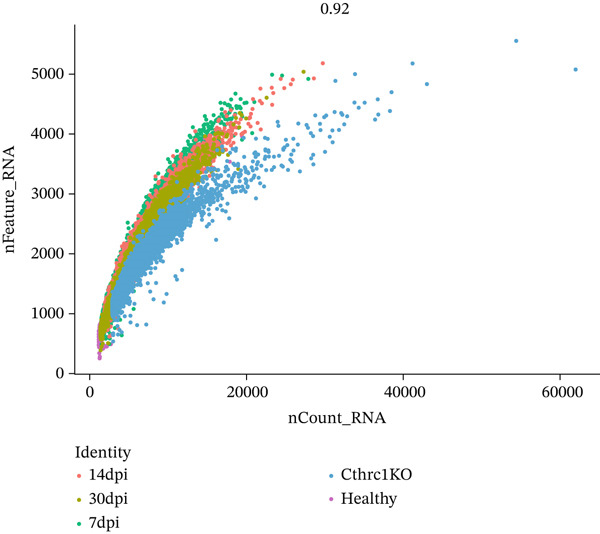
(b)

**Figure:**
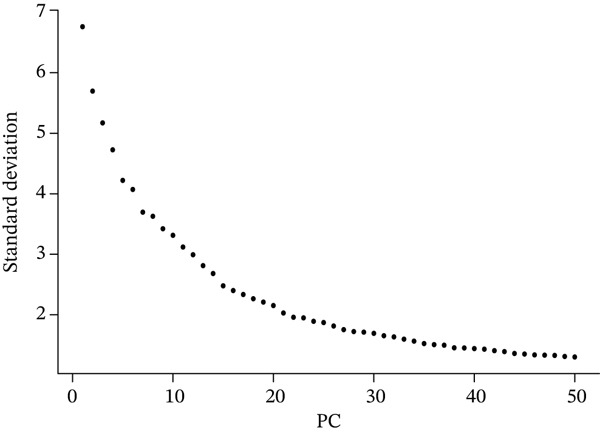
(c)

**Figure:**
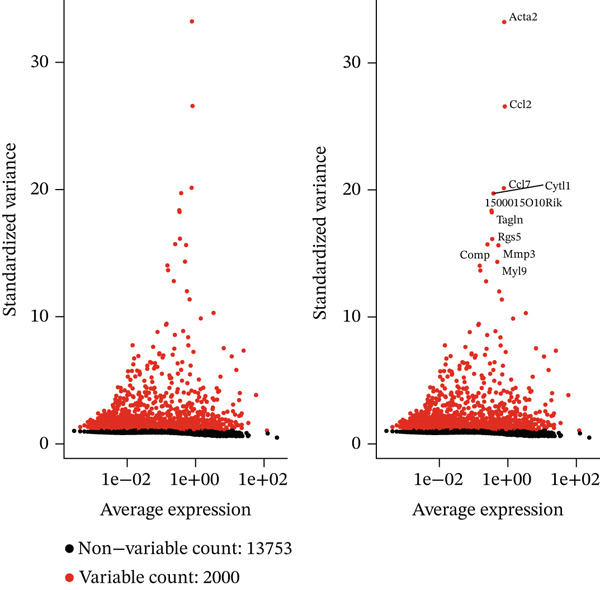
(d)

### 3.2. PCA and Gene Expression Patterns in MI Model

A multifaceted examination of gene expression profiles characterizes the MI model in this figure. Gene loadings for PC_1 and PC_2 appear in Figure 2a, exposing critical genes that propel transcriptional variation throughout the dataset. PC_1 draws its major contributions from Mfap5, S100a10, Lyz2, and CD34, whereas Gpx3, Hsp11l1, Ehd3, and Cryab2 predominantly shape PC_2. These identified genes underscore significant biological processes operating within cardiac remodeling following injury. Statistical representation of principal components with corresponding *p* values features in Figure 2b, where the first 15 PCs consistently achieve exceptional statistical significance (*p* < 1.0e − 102), thereby confirming robust cellular population separation based on transcriptomic signatures. Validation of the identified components′ statistical integrity comes from the theoretical versus empirical distribution curve. Figure 2c delivers a hierarchical clustering heatmap spanning different samples, unveiling distinct expression patterns that separate into well‐defined clusters. Through a color gradient transitioning from blue (indicating low expression) to red (signifying high expression), coordinated gene regulation events become visible, potentially signifying diverse cell types or states throughout the cardiac infarction response. Molecular mechanisms and cellular heterogeneity underlying myocardial injury and repair processes receive valuable clarification from these combined analyses, which pinpoint potential therapeutic targets aimed at enhancing cardiac recovery.

Figure 2Principal component analysis and gene expression patterns in myocardial infarction model. (a) Gene loadings for PC_1 and PC_2, revealing key genes driving transcriptional variation in the dataset. (b) Statistical representation of principal components with their associated *p* values, demonstrating that the first 15 PCs all reach high statistical significance. (c) Hierarchical clustering heatmap of gene expression across different samples, revealing distinct expression patterns that segregate into clear clusters.

**Figure:**
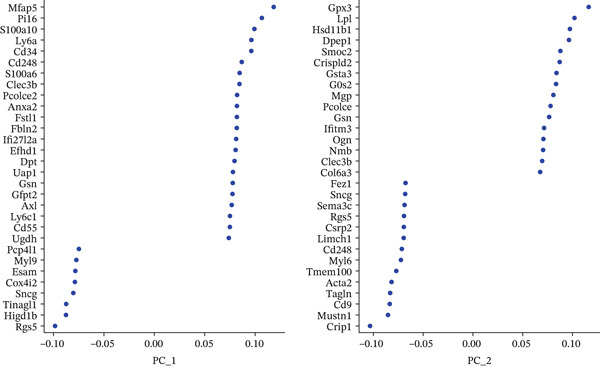
(a)

**Figure:**
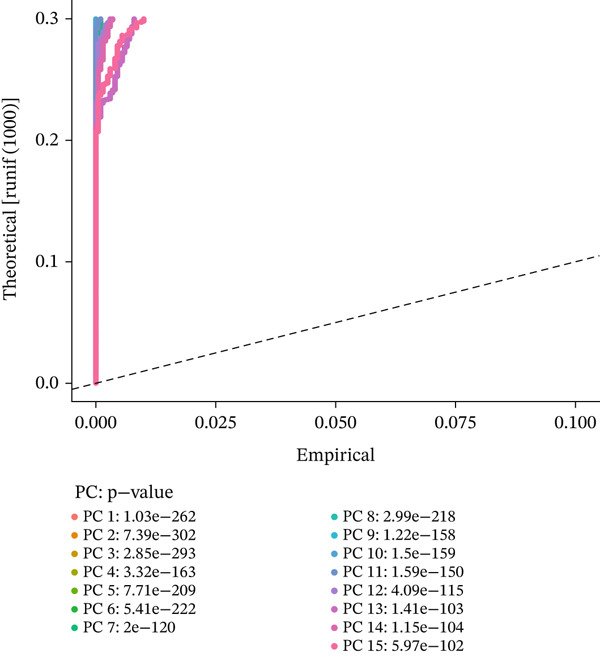
(b)

**Figure:**
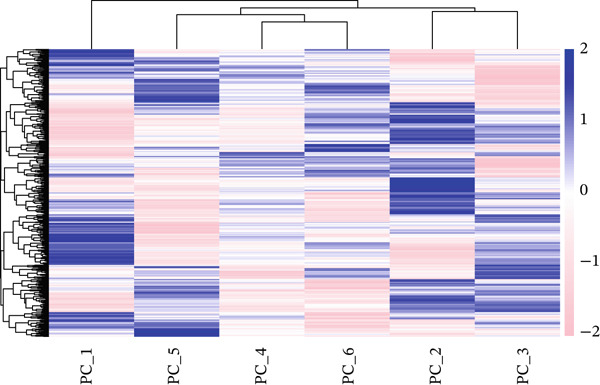
(c)

### 3.3. Cellular Heterogeneity and Population Dynamics in the MI Model

Through dimensionality‐reduction analyses, this figure depicts the single‐cell transcriptomic landscape alongside cellular diversity characterizing the MI model. UMAP visualizations occupy Panels a and b, displaying cellular populations through unsupervised clustering colors (Figure 3a) and annotated cell type designations (Figure 3b). Multiple distinct cell populations participating in cardiac injury response gain identification, encompassing fibroblasts, immune cells (neutrophils, macrophages, and monocytes), endothelial cells, cardiomyocytes, lymphocytes, and vascular smooth muscle cells. Well‐defined clusters with clear boundaries characterize each cell type, mirroring their distinctive transcriptional profiles. Alternative dimensionality reduction utilizing t‐SNE manifests in Figure 3c,d, providing additional validation for cell type segregation observed within UMAP plots, albeit with modified spatial arrangements. The robustness of the identified cellular populations gains confirmation through consistency between UMAP and t‐SNE visualizations. Immune cell populations (especially macrophages and neutrophils) demonstrate prominence within the dataset, implying their critical function in inflammatory and repair processes succeeding MI. A comprehensive perspective of cellular interactions driving cardiac remodeling emerges from the presence of both resident cardiac cells and infiltrating immune populations. The complex cellular ecosystem arising during postinfarction recovery receives emphasis through these findings, presenting potential targets for therapeutic interventions designed to optimize healing processes.

Figure 3Cellular heterogeneity and population dynamics in the myocardial infarction model. UMAP (uniform manifold approximation and projection) visualizations of the cellular populations colored by (a) unsupervised clustering and (b) annotated cell types. (c, d) Alternative dimensionality reduction using t‐SNE (t‐distributed stochastic neighbor embedding), which further validates the cell type segregation observed in the UMAP plots but with slightly different spatial arrangements.

**Figure:**
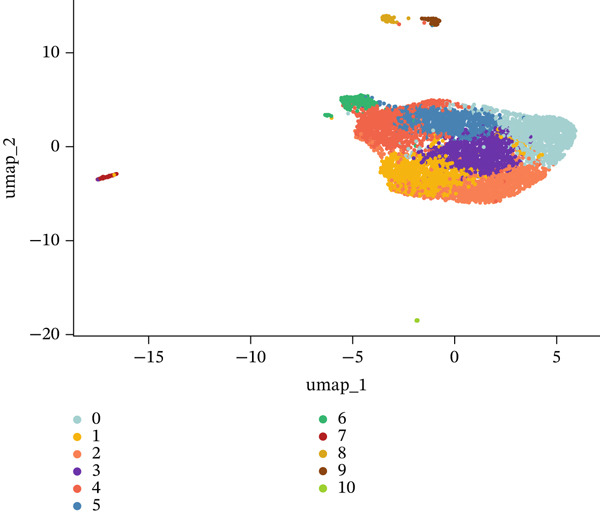
(a)

**Figure:**
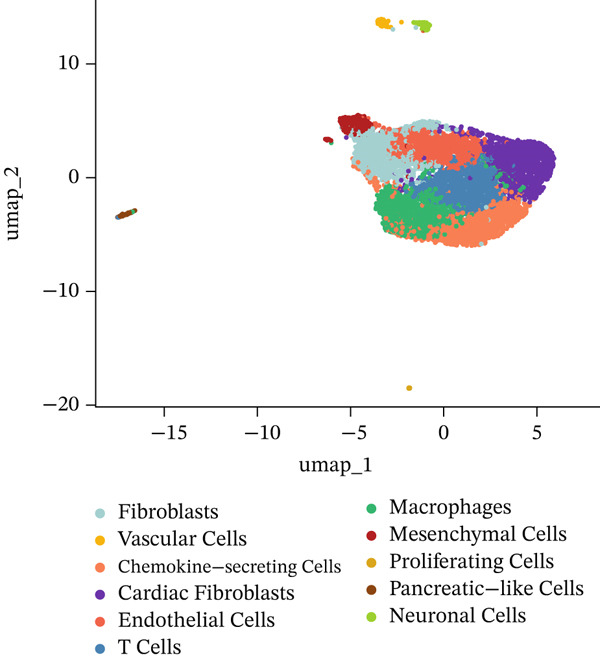
(b)

**Figure:**
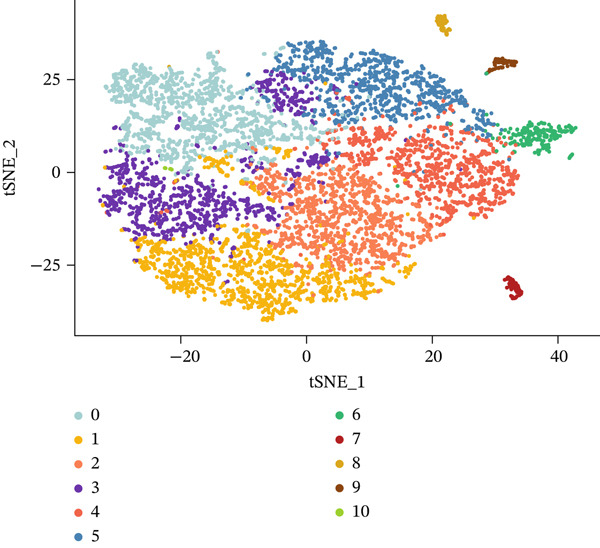
(c)

**Figure:**
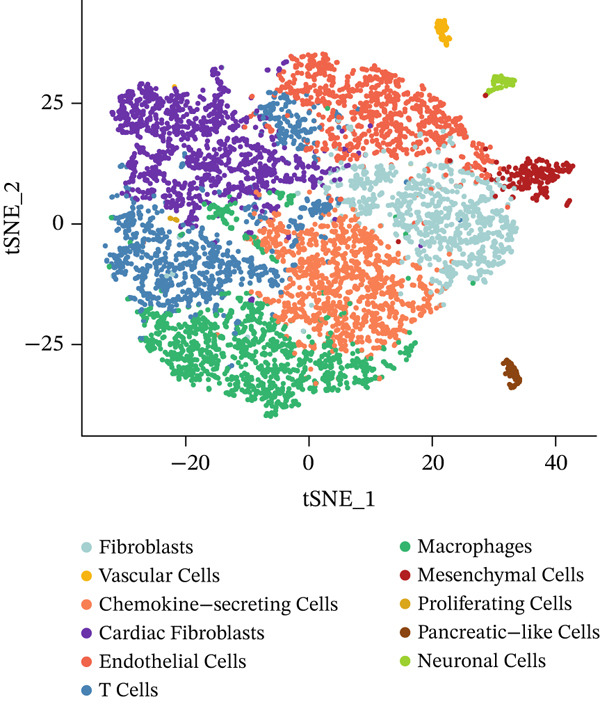
(d)

### 3.4. Cellular Composition and Intercellular Communication Networks in the MI Model

Cellular proportions and cell–cell communication networks in the MI model receive comprehensive examination within this figure. Pie charts in Figure 4a portray cellular composition across varying conditions, demonstrating pronounced shifts in cell type proportions throughout cardiac healing. Fibroblasts (pink), immune cells (purple and teal), and other cardiac resident cells (green) constitute the predominant cell populations, exhibiting noteworthy changes in relative abundances responding to myocardial injury. Global intercellular communication networks occupy Figure 4b,c, wherein nodes signify different cell types while connecting lines denote ligand–receptor interactions. Connection thickness and color intensity reflect cellular crosstalk strength, emphasizing key cellular hubs that orchestrate inflammatory and repair responses. Specific intercellular communication networks involving individual cell types receive focused attention in Figures 4d, 4e, 4f, and 4g—fibroblasts (Figure 4d), endothelial cells (Figure 4e), cardiomyocytes (Figure 4f), and macrophages (Figure 4g). Unique communication patterns for each cell type emerge from these focused networks, with macrophages exhibiting particularly extensive connectivity that suggests their central coordinating role in postinfarction response. Strong interactions between fibroblasts and both cardiomyocytes and immune cells become apparent, likely reflecting their participation in scar formation and matrix remodeling. Significant communication between endothelial cells and immune populations becomes evident, potentially connecting to vascular repair and inflammatory cell recruitment. Primary connections with fibroblasts and macrophages characterize cardiomyocyte interactions, highlighting crucial relationships for cardiac regeneration. Intricate cellular interplay driving myocardial repair gains valuable illumination through these collective findings, which identify potential therapeutic targets for modulating post‐MI healing processes.

Figure 4Cellular composition and intercellular communication networks in the myocardial infarction model. (a) Pie charts illustrating the cellular composition across different conditions, showing distinct shifts in cell type proportions during cardiac healing. (b, c) Global intercellular communication networks, where nodes represent different cell types and connecting lines indicate ligand–receptor interactions. Specific intercellular communication networks involving individual cell types—fibroblasts (d), endothelial cells (e), cardiomyocytes (f), and macrophages (g).

**Figure:**
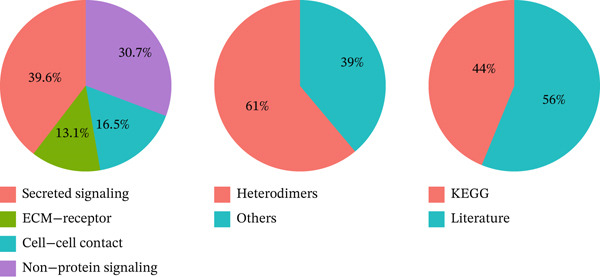
(a)

**Figure:**
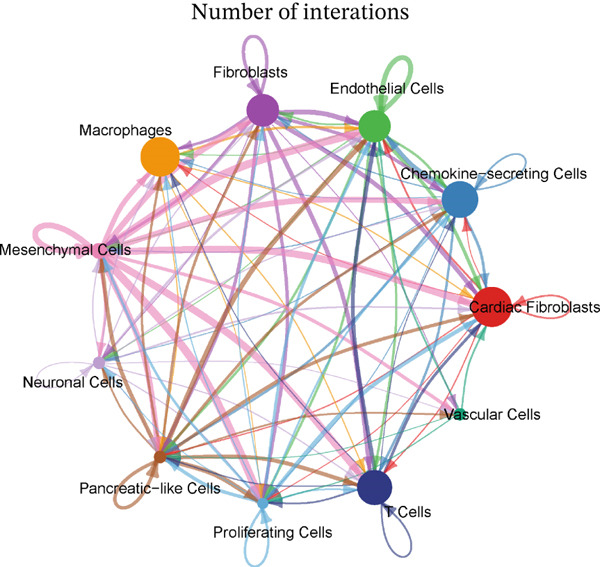
(b)

**Figure:**
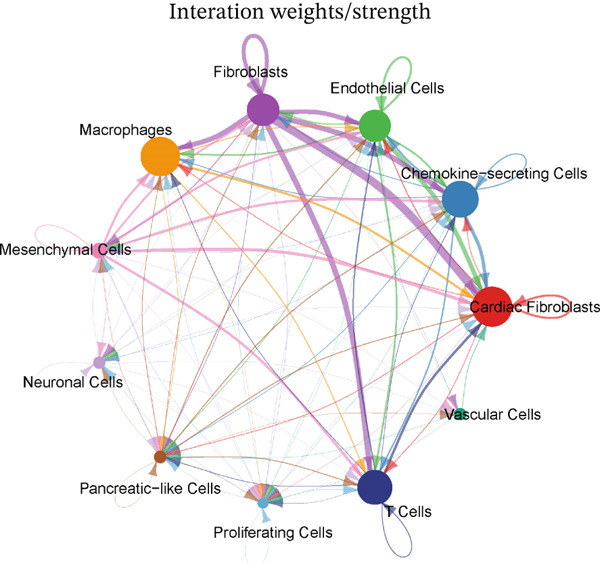
(c)

**Figure:**
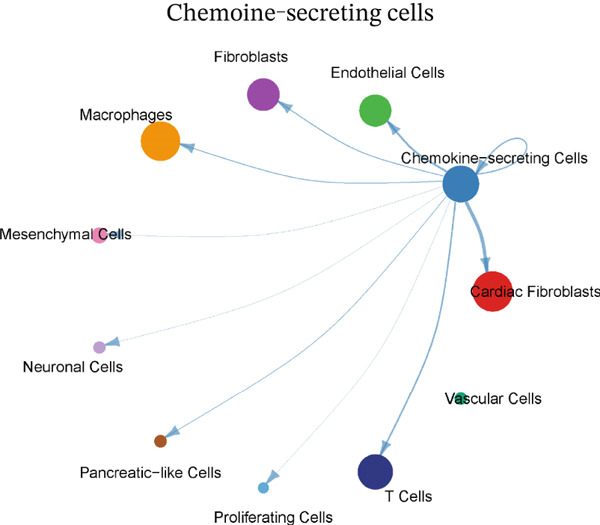
(d)

**Figure:**
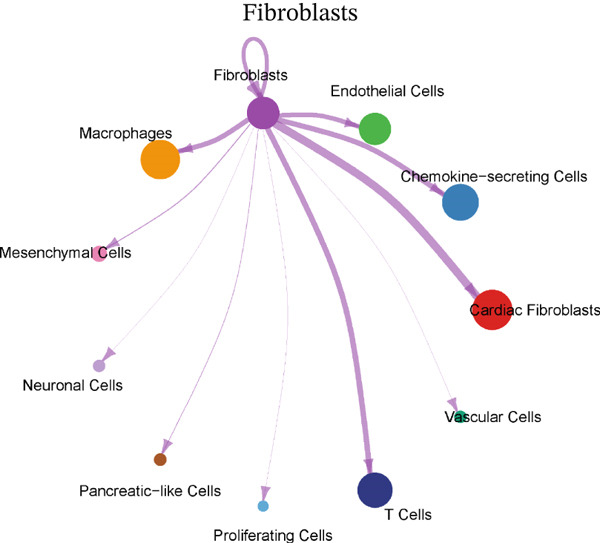
(e)

**Figure:**
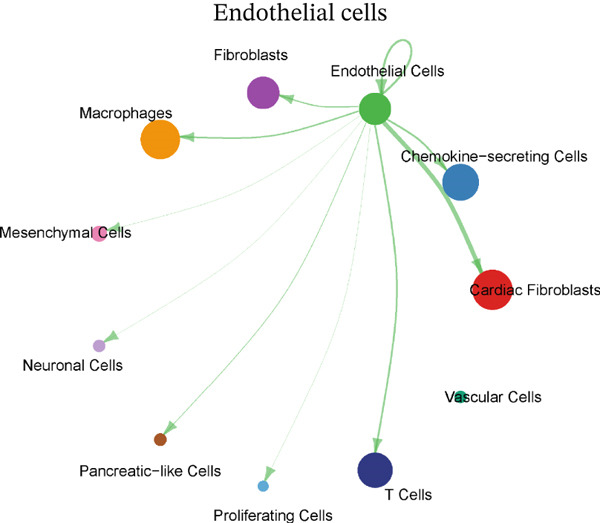
(f)

**Figure:**
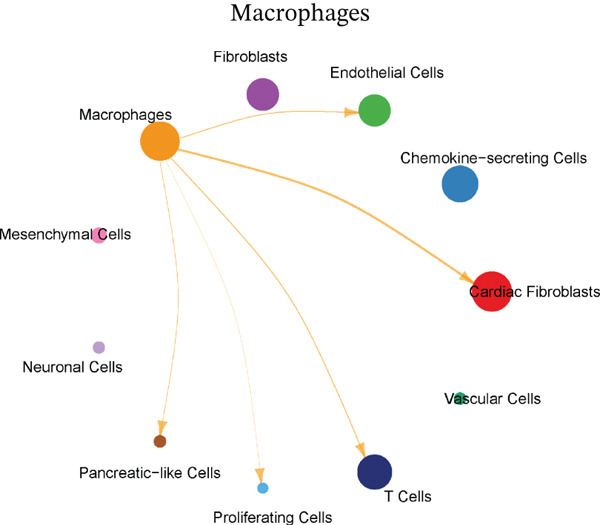
(g)

### 3.5. MIF Signaling Network Analysis in the MI Microenvironment

The MIF signaling pathway network receives elucidation within the MI context through this figure. A comprehensive visualization of the MIF signaling network appears in Figure 5a, depicting source and target cell interactions. Chemokine‐secreting cells and cardiac fibroblasts emerge as primary MIF signal sources (left side), while these identical cell types, alongside mesenchymal cells and neuronal cells, operate as principal target cells (right side). Signaling strengths between different cell populations gain representation through colored lines, revealing particularly robust connections between macrophages and cardiac fibroblasts. A heatmap quantifying communication strengths between cell types features in Figure 5b, where darker coloration indicates stronger interactions. Chemokine‐secreting cells, cardiac fibroblasts, vascular cells, and macrophages function as the predominant MIF signaling contributors, while cardiac fibroblasts, endothelial cells, and mesenchymal cells serve as major signal recipients, according to this analysis. The strongest interactions materialize between chemokine‐secreting cells and cardiac fibroblasts, as the heatmap reveals, implying a crucial regulatory axis within the postinfarction microenvironment. Violin plots in Figure 5c display expression distribution of MIF and its receptor Ackr3 spanning different cell populations. Widespread distribution of MIF expression characterizes all analyzed cell types, though with varying intensities, signifying its ubiquitous function in cardiac healing processes. Conversely, Ackr3 expression demonstrates primary restriction to cardiac fibroblasts, endothelial cells, and proliferating cells, indicating selective MIF signal reception. Intricate MIF‐mediated intercellular communication networks governing inflammatory and repair responses following MI gain valuable insight through these collective findings, which identify potential therapeutic targets for modulating cardiac healing and preventing adverse remodeling.

Figure 5MIF signaling network analysis in the myocardial infarction microenvironment. (a) Comprehensive visualization of the MIF signaling network, illustrating source and target cell interactions. (b) Heatmap quantifying the communication strengths between cell types, with darker colors indicating stronger interactions. (c) Violin plots showing the expression distribution of MIF and its receptor Ackr3 across different cell populations.

**Figure:**
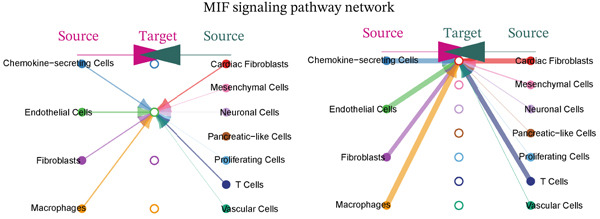
(a)

**Figure:**
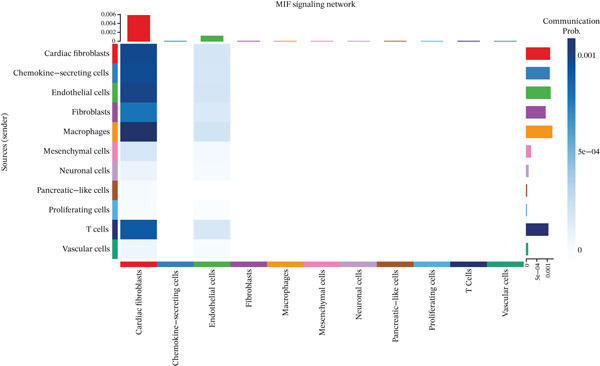
(b)

**Figure:**
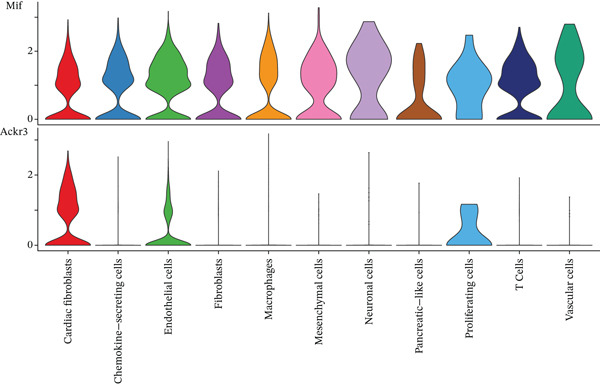
(c)

### 3.6. Pseudotime Trajectory Analysis of Cellular Dynamics Following MI

Developmental paths and transitions of various cell types during MI recovery receive comprehensive revelation through this figure′s pseudotime trajectory analysis. A pseudotime trajectory plot with cells colored according to developmental states (numbered 1–8) occupies Figure 6a, depicting a continuous progression along a curved path. From early response cells at one terminus to mature phenotypes at the opposite end, this trajectory demonstrates a clear developmental continuum, with transitional states arranged sequentially along the curve. The same trajectory colored by pseudotime value (dark blue to light blue gradient) features in Figure 6b, confirming directional progression of cellular states while highlighting temporal dynamics throughout the cardiac healing process. Cell type information overlay on the trajectory appears in Figure 6c, with different colors representing distinct cell populations including cardiac fibroblasts, chemokine‐secreting cells, macrophages, endothelial cells, mesenchymal cells, neuronal cells, proliferating cells, pancreatic‐like cells, vascular cells, and T cells. How different cell types distribute along the developmental continuum becomes visible through this visualization, with immune cells predominantly positioned at early pseudotime points and tissue‐resident cells at later stages, reflecting the transition from inflammatory to repair phases. A branched trajectory analysis showing hierarchical relationships between cell populations features in Figure 6d, with multiple bifurcation points indicating critical cell fate decision events. Progressive specialization from common progenitor states into distinct functional subtypes during cardiac healing response receives suggestion through this branched structure. Phenotypic plasticity and diverse roles throughout the repair process gain emphasis through certain cell populations, particularly fibroblasts and immune cells, appearing at multiple branch points. Cellular reprogramming and differentiation dynamics driving postinfarction myocardial repair gain valuable insight through these collective findings, which identify potential intervention points for therapeutic modulation of the healing trajectory.

Figure 6Pseudotime trajectory analysis of cellular dynamics following myocardial infarction. (a) Pseudotime trajectory plot with cells colored according to their developmental states (numbered 1–8), illustrating a continuous progression along a curved path. (b) The same trajectory colored by pseudotime value (dark blue to light blue gradient), confirming the directional progression of cellular states and highlighting temporal dynamics during the cardiac healing process. (c) Cell type information overlaid on the trajectory, with different colors representing distinct cell populations including cardiac fibroblasts and chemokine‐secreting cells. (d) Branched trajectory analysis showing the hierarchical relationships between cell populations, with multiple bifurcation points indicating critical cell fate decision events.

**Figure:**
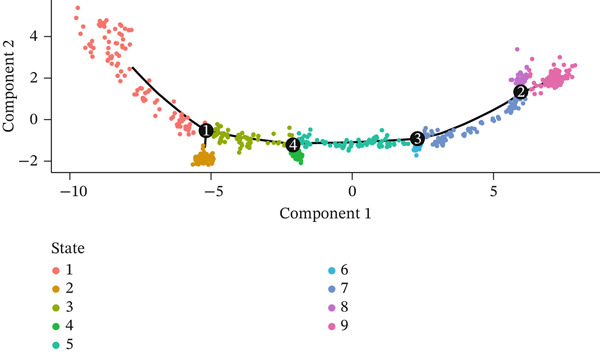
(a)

**Figure:**
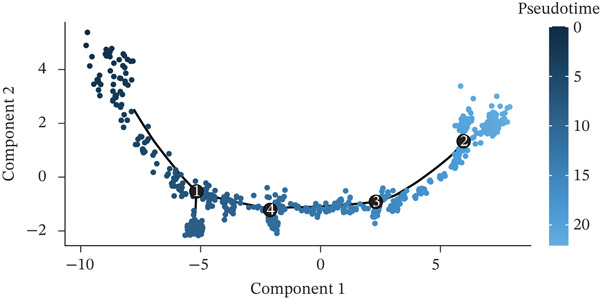
(b)

**Figure:**
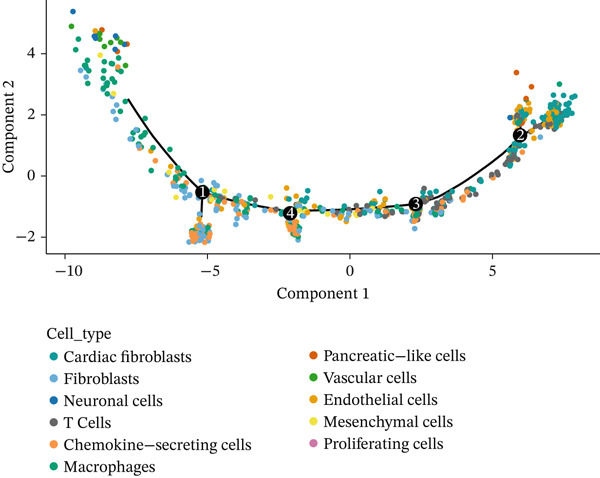
(c)

**Figure:**
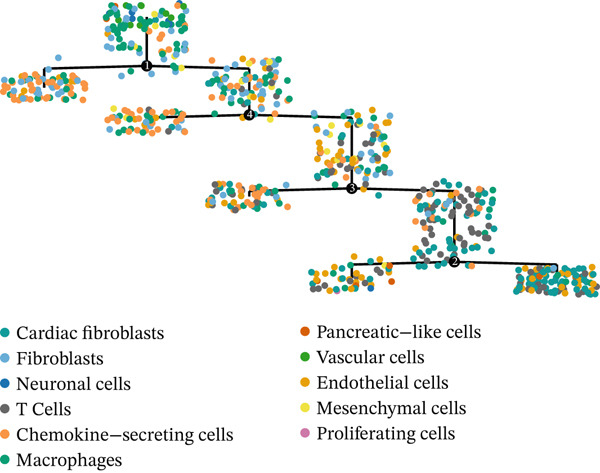
(d)

### 3.7. Pseudotime Distribution Analysis and State‐Specific Gene Expression Dynamics in MI Recovery

Cell type distribution along the pseudotime trajectory alongside expression dynamics of key regulatory genes during MI recovery receives detailed analysis in this figure. Density distribution of different cell populations across the pseudotime continuum appears in Figure 7a, unveiling distinct temporal patterns of cellular activity. Prominent presence at early pseudotime points characterizes vascular cells (green) and endothelial cells (yellow), implying their early activation following cardiac injury. Distribution across intermediate timepoints describes macrophages and chemokine‐secreting cells, reflecting their sustained role throughout inflammatory and early repair phases. Notably, a marked peak at later pseudotime points distinguishes proliferating cells (pink), signifying enhanced proliferative activity during advanced cardiac repair stages. Insights into the coordinated cellular response orchestrating postinfarction healing emerge from this temporal organization of cell types. Expression patterns of four pivotal genes (Atpdv1h, Lypla1, Mrpl15, and Tcea1) across different cellular states and pseudotime progression receive examination in Figure 7b,c. Violin plots of gene expression across nine distinct cellular states (colored from red to pink) occupy Panel b, unveiling state‐specific expression patterns. Scatter plots of the same genes with expression levels plotted against pseudotime, colored by cellular state, feature in Panel c. A gradual increase along pseudotime, particularly in States 6–9, characterizes Atpdv1h expression, suggesting its importance in later repair phases. Elevated expression in intermediate states (3–4) describes Lypla1, while a more complex pattern with peaks in States 3 and 8 distinguishes Mrpl15 expression. Progressively increasing expression along pseudotime, with the highest levels in final states, characterizes Tcea1 display. Temporal dynamics of different cell populations and their associated gene expression programs during cardiac healing gain valuable insight through these collective findings, identifying potential molecular drivers for each recovery phase and suggesting targeted therapeutic opportunities for enhancing myocardial repair. Table S1 provides a complete gene list.

Figure 7Pseudotime distribution analysis and state‐specific gene expression dynamics in myocardial infarction recovery. (a) Density distribution of different cell populations across the pseudotime continuum, revealing distinct temporal patterns of cellular activity. (b, c) Expression patterns of four pivotal genes (Atpdv1h, Lypla1, Mrpl15, and Tcea1) across different cellular states and pseudotime progression.

**Figure:**
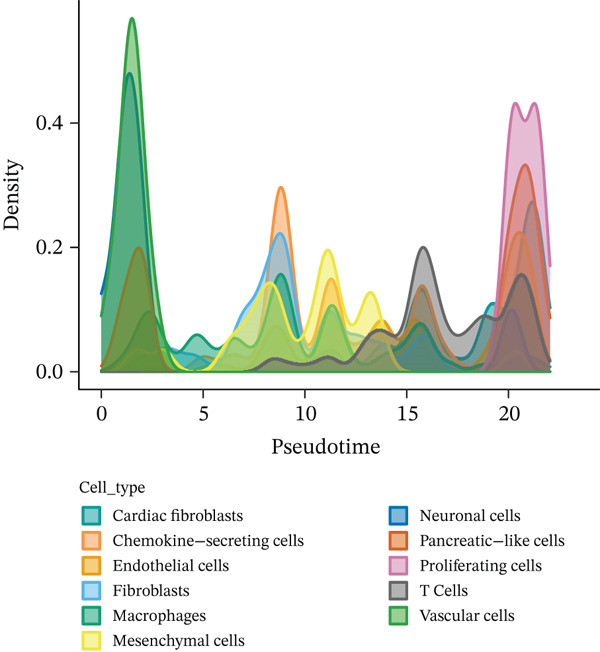
(a)

**Figure:**
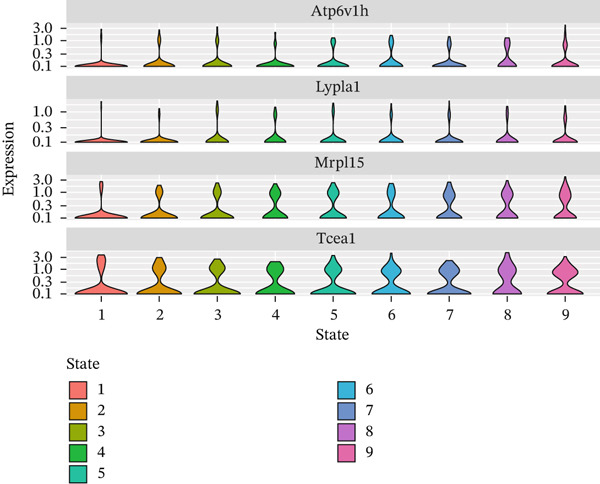
(b)

**Figure:**
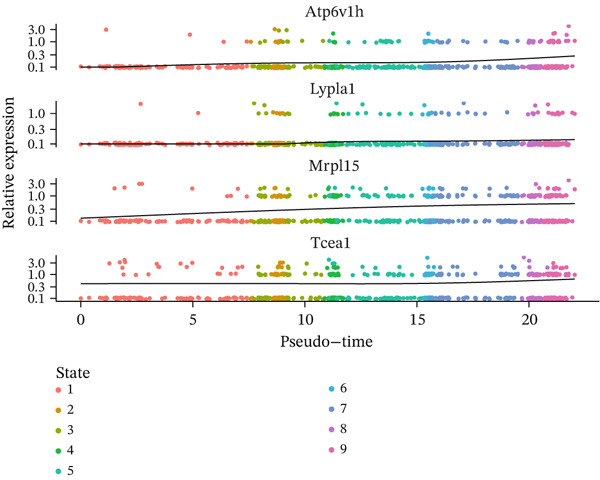
(c)

### 3.8. Temporal Gene Expression Patterns and Branch‐Specific Dynamics in Cardiac Repair

Gene expression dynamics across pseudotime and cell fate branches during MI recovery receive a comprehensive analysis in this figure. A hierarchically clustered heatmap of gene expression patterns along pseudotime occupies Figure 8a, unveiling four distinct modules of coregulated genes. Gradual activation as pseudotime progresses characterizes the top two modules, likely representing genes involved in cardiac repair and tissue remodeling processes. In opposition, declining expression along pseudotime distinguishes the bottom two modules, potentially indicating inflammatory or acute response genes that diminish as healing advances. Coordinated transition from inflammatory to reparative phases during cardiac healing receives reflection through this temporal organization of gene programs. A similar heatmap but separating expression patterns by developmental branches features in Figure 8b, highlighting branch‐specific gene regulation. Unique transcriptional signatures associated with distinct cell fate decisions gain revelation through this analysis, with certain gene clusters showing branch‐selective expression patterns. Differentiation of cells toward specialized phenotypes required for different repair process aspects likely receives guidance from these branch‐specific programs. Scatter plots tracking the expression of six key genes (Apoa, Cldn1, Dpep1, Map, and P16) across pseudotime with points colored by cell type and shapes indicating different developmental branches occupy Figure 8c. Distinct expression dynamics characterize these genes, with some showing branch‐specific patterns and others exhibiting universal trends across lineages. Important regulators involved in lipid metabolism (Apoa), cell adhesion (Cldn1), peptide metabolism (Dpep1), cytoskeletal organization (Map), and cell cycle regulation (P16) receive representation through these genes, notably. Divergent molecular mechanisms directing different cell fate trajectories during cardiac repair receive suggestion through the branch‐specific expression of certain genes, particularly pronounced in Branch Y_53 versus Y_9. Temporal and lineage‐specific gene expression programs orchestrating postinfarction myocardial recovery gain valuable insight through these collective findings, identifying potential molecular targets for therapeutic intervention to optimize the healing process.

Figure 8Temporal gene expression patterns and branch‐specific dynamics in cardiac repair. (a) Hierarchically clustered heatmap of gene expression patterns along pseudotime, revealing four distinct modules of coregulated genes. The top two modules show gradual activation as pseudotime progresses, likely representing genes involved in cardiac repair and tissue remodeling processes. (b) Similar heatmap separating expression patterns by developmental branches, highlighting branch‐specific gene regulation. (c) Scatter plots tracking the expression of six key genes (Apoa, Cldn1, Dpep1, Map, and P16) across pseudotime with points colored by cell type and shapes indicating different developmental branches. These genes display distinct expression dynamics, with some showing branch‐specific patterns and others exhibiting universal trends across lineages.

**Figure:**
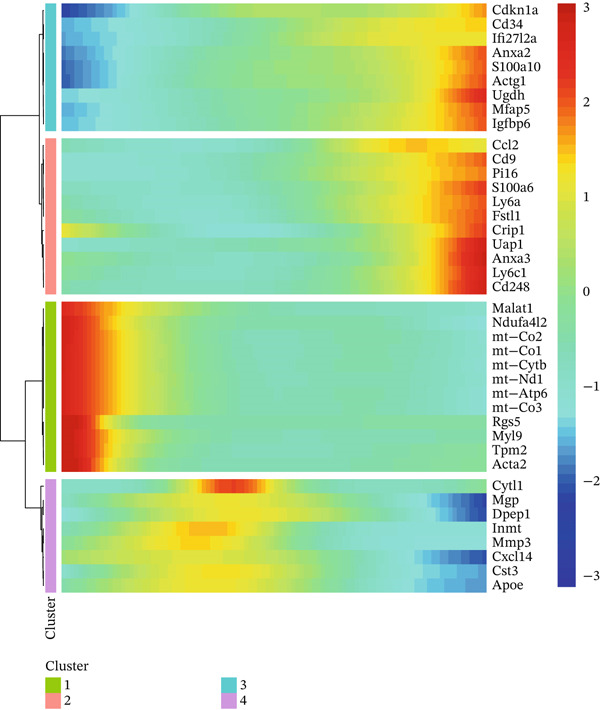
(a)

**Figure:**
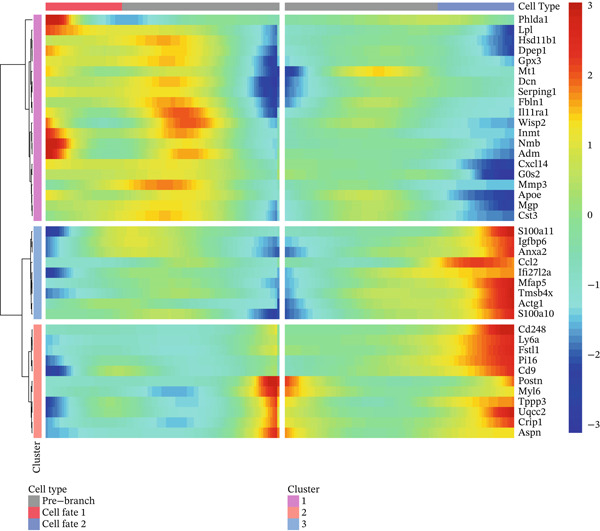
(b)

**Figure:**
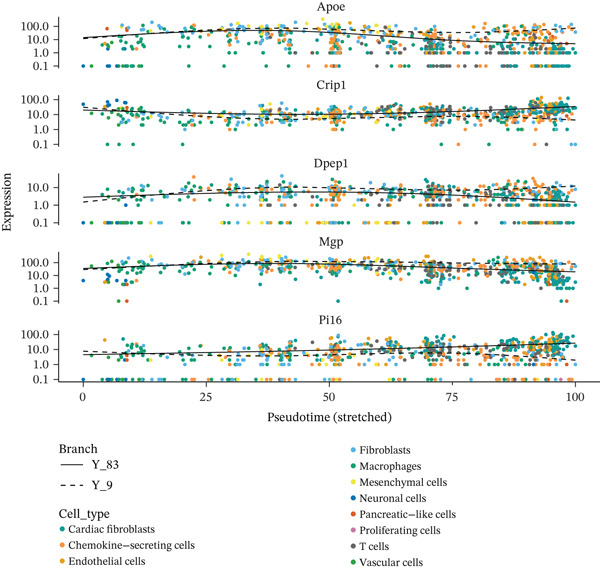
(c)

## 4. Discussion

A comprehensive single‐cell transcriptomic analysis of MI recovery emerges from the present study, unveiling the dynamic cellular landscape alongside molecular mechanisms that govern cardiac repair. Through the integration of multiple computational approaches, we have assembled a high‐resolution map depicting cellular transitions, intercellular communication networks, and temporally regulated gene expression programs in the aftermath of MI. Our understanding of the complex and coordinated cardiac repair process receives significant enhancement from our findings, which additionally propose potential therapeutic targets for improving outcomes in MI patients.

Diverse cell populations participating in cardiac repair received identification through our analysis, confirming the complex cellular ecosystem arising following MI. Previous findings gain extension through the discovery of distinct fibroblast, endothelial, and macrophage subpopulations bearing unique transcriptional signatures, emphasizing the considerable heterogeneity existing within these broadly defined cell types. Of particular note was specialized chemokine‐secreting cells′ identification, which appear to execute crucial roles in orchestrating inflammatory and repair responses through paracrine signaling mechanisms.

A continuous progression of cellular states during cardiac repair became evident through pseudotime trajectory analysis, transitioning from inflammatory to proliferative and ultimately to maturation phases. Important insights into developmental relationships between different cell states emerge from the organization of cells along this trajectory. Early‐responding immune cells followed by reparative mesenchymal populations align with established temporal phases of cardiac healing, though offering unprecedented resolution of transitional states between these phases. Critical cell fate decision points receive further illumination through the branched trajectory structure, implying that therapeutic interventions targeting these branch points might effectively modulate the repair process toward more favorable outcomes.

Particular interest arose from the distinct distribution of cell types along the pseudotime continuum. Prominent presence at early pseudotime points characterized vascular and endothelial cells, implying their early activation following cardiac injury, potentially supporting revascularization of damaged tissue. In contrast, later pseudotime points witnessed proliferating cells reaching their peak, corresponding to the proliferative phase of cardiac repair when active tissue regeneration and remodeling occur. A framework for understanding sequential cellular events driving cardiac repair emerges from this temporal organization of cell types, potentially informing the timing of targeted therapeutic interventions.

The MIF signaling pathway gained identification through our analysis as a central mediator of intercellular communication during cardiac repair. A complex autocrine and paracrine signaling network regulating the repair process receives suggestion from the finding that chemokine‐secreting cells and cardiac fibroblasts serve as primary MIF signal sources, while these same cell types along with mesenchymal and neuronal cells function as principal targets.

Our analysis identified the MIF signaling pathway as a central mediator of intercellular communication during cardiac repair. The finding that chemokine‐secreting cells and cardiac fibroblasts serve as primary MIF signal sources, while these same cell types along with mesenchymal and neuronal cells function as principal targets, suggests a complex autocrine and paracrine signaling network regulating the repair process.

A specialized role for this signaling axis gains further support from the differential expression pattern of MIF and its receptor Ackr3. Broad distribution across various cell types characterized MIF expression, while Ackr3 expression demonstrated primary restriction to cardiac fibroblasts, endothelial cells, and proliferating cells [21–23]. The targeted effects of MIF signaling on specific cell populations during cardiac repair may receive explanation through this selective receptor expression. Previous studies have implicated MIF in cardioprotection following ischemic injury, yet our findings deliver the first comprehensive map of MIF‐mediated cellular communication in the post‐MI heart.

Particular importance for regulating fibroblast activation and function during repair may characterize the strong connection between chemokine‐secreting cells and cardiac fibroblasts in the MIF signaling network [24]. Extensive and dynamic crosstalk between diverse cardiac cell populations became evident through our analysis of global cell–cell communication networks, beyond the MIF pathway. Not only their importance as effector cells in the inflammatory response but also their role as coordinators of the overall repair process through interactions with various cardiac cell types receives underscore from the identification of macrophages as central hubs in these communication networks.

A cooperative relationship likely shaping fibroblast activation and extracellular matrix production during repair receives suggestion from the strong bidirectional communication between macrophages and fibroblasts. Similarly, potential mechanisms coordinating inflammatory cell recruitment and angiogenesis gain highlight through the significant interactions between endothelial cells and immune populations. Important signaling events governing cardiomyocyte survival and functional adaptation following injury likely receive reflection from the connections between cardiomyocytes and both fibroblasts and macrophages.

Alignment with emerging concepts of immune cells as central regulators of tissue repair characterizes these findings, yet providing unprecedented detail on the specific communication pathways mediating these effects in the cardiac context. Potential targets for therapeutic intervention to modulate specific aspects of the repair process while minimizing unintended effects on other cellular interactions emerge from the identification of key cellular communication hubs and pathways.

Distinct modules of coregulated genes with specific temporal patterns during cardiac repair received revelation through our analysis of gene expression dynamics across pseudotime. Insights into the molecular drivers of the transition from inflammatory to reparative phases emerge from the identification of genes that undergo progressive activation or repression along the pseudotime trajectory. Potential importance in advanced repair processes receives suggestion from the gradual increase in expression of genes like Atpdv1h in later states, while the intricate regulation of cellular functions across different repair phases gains highlight through the complex expression patterns of genes like Mrpl15.

Particularly intriguing was branch‐specific gene expression patterns′ identification, implying divergent molecular mechanisms directing different cell fate trajectories. Specific molecular programs guiding cellular specialization during repair receive indication from the differential expression of genes involved in lipid metabolism (Apoa), cell adhesion (Cldn1), peptide metabolism (Dpep1), cytoskeletal organization (Map), and cell cycle regulation (P16) across developmental branches. The necessary diversification of cellular phenotypes required to accomplish the various tasks of cardiac repair, from inflammation resolution to matrix deposition and tissue remodeling, likely receives reflection through these branch‐specific programs.

Complex and context‐dependent roles in cardiac repair, regulating cardiomyocyte survival, cardiac progenitor cell activation, and fibroblast function, characterize the Wnt signaling pathway. Intriguing connections between MIF signaling and Wnt pathway activity emerged through our analysis. Specifically, correlated expression patterns with MIF targets across our pseudotime trajectory characterized Wnt target genes (including Axin2, Lef1, and Tcf7).

Demonstration that MIF can modulate Wnt signaling through multiple mechanisms comes from recent studies: (1) MIF‐mediated regulation of glycogen synthase kinase‐3*β* (GSK‐3*β*) activity, a key negative regulator of canonical Wnt signaling; (2) direct interaction between MIF receptors (CD74/CD44) and Wnt coreceptors (LRP5/6) at the cell membrane; and (3) epigenetic regulation of Wnt pathway components through MIF‐dependent chromatin remodeling [25]. Partial mediation through modulation of Wnt pathway activity may characterize the beneficial effects of MIF signaling in cardiac repair, according to these converging lines of evidence. A novel regulatory mechanism in cardiac repair that warrants further investigation and may offer additional therapeutic opportunities for enhancing post‐MI recovery gains representation through this crosstalk between MIF and Wnt signaling pathways.

Multiple aspects of the repair process could receive influence from modulation of this pathway, as the elucidation of the MIF signaling network suggests. Selective modulation of MIF signaling in cardiac fibroblasts and endothelial cells while sparing other cell types might become achievable through targeting this receptor, potentially reducing off‐target effects, given the restricted expression of the MIF receptor Ackr3 to specific cell populations [26, 27]. Synergistic effects in promoting cardiac repair and preventing adverse remodeling might receive achievement from combinatorial therapeutic approaches targeting both pathways simultaneously, as the identified crosstalk between MIF and Wnt signaling pathways suggests.

Further support for the concept of coordinated transcriptional programs driving the repair process emerges from the hierarchical clustering of gene expression patterns along pseudotime, revealing four distinct modules of coregulated genes. Elegant capture of the molecular basis of the inflammatory‐to‐reparative transition that proves critical for successful cardiac healing emerges from the gradual activation of genes likely involved in tissue remodeling juxtaposed with the declining expression of inflammatory response genes.

Several important implications for developing novel therapeutic strategies to enhance cardiac repair following MI arise from our findings. Potential targets for interventions aimed at promoting beneficial cell phenotypes while inhibiting detrimental ones receive provision through the detailed characterization of cellular states and transitions. For instance, guidance of cell fate decisions toward more regenerative phenotypes rather than excessive fibrosis might receive achievement from therapeutics targeting the identified branch points in cellular trajectories. Multiple aspects of the repair process could receive influence from modulation of this pathway, as the elucidation of the MIF signaling network suggests. Selective modulation of MIF signaling in cardiac fibroblasts and endothelial cells while sparing other cell types might become permissible through targeting this receptor, potentially reducing off‐target effects, given the restricted expression of the MIF receptor Ackr3 to specific cell populations [26, 27]. The potential of macrophage‐directed therapies to influence overall repair outcomes receives highlight from the central role of macrophages in coordinating the repair response. Effective reshaping of the repair environment to favor functional recovery over adverse remodeling might emerge from strategies aimed at modulating macrophage phenotype or specific aspects of macrophage‐mediated intercellular communication. Suggestions that different molecular pathways should be targeted at specific phases of the repair process for optimal effect could emerge from the temporal dynamics of gene expression identified in this study. Potential molecular targets for stage‐specific interventions receive provision through the identification of key regulatory genes with dynamic expression across pseudotime.

## 5. Limitations and Future Directions

Several limitations should be acknowledged despite the comprehensive nature of our analysis. First, only a snapshot of the transcriptome receives capture through scRNA‐seq, which provides unprecedented resolution of cellular heterogeneity, and important posttranscriptional and posttranslational regulatory events may be missed. A more complete picture of the molecular landscape during cardiac repair would receive provision through integration with proteomics and epigenomic approaches. Second, primary focus on cellular dynamics within the infarcted region and border zone characterized our analysis, potentially missing important events in remote myocardium that could influence global cardiac function. Better understanding of the regional heterogeneity of repair processes across the infarcted heart should receive incorporation through future studies employing spatial transcriptomic approaches.

## 6. Conclusion

In conclusion, a comprehensive single‐cell transcriptomic atlas of the cardiac repair process following MI receives provision from this study, unveiling unprecedented details about cellular heterogeneity, developmental trajectories, intercellular communication networks, and temporal gene expression programs. The central role of macrophages in orchestrating repair, the importance of the MIF signaling pathway in mediating intercellular communication, and the existence of distinct molecular programs guiding cell fate decisions during different phases of repair gain highlight from our findings.

## Author Contributions

Jianfeng Zhao and Junhui Gong contributed equally to this work.

## Funding

No funding was received for this manuscript.

## Ethics Statement

The authors have nothing to report.

## Conflicts of Interest

The authors declare no conflicts of interest.

## Supporting information


**Supporting Information** Additional supporting information can be found online in the Supporting Information section. Detailed sequencing quality control indicators for samples.

## Data Availability

The data that support the findings of this study are available from the corresponding author upon reasonable request.
