# Supplementary material for: Single‐Cell Transcriptomics Reveals Dynamic Cellular Interactions and Molecular Mechanisms in Myocardial Infarction Recovery
Source: Int J Genomics. 2026 Mar 6;2026:4888573. doi: 10.1155/ijog/4888573 (PMC12966613; doi:10.1155/ijog/4888573)
Supplement: Supplementary file 1 — Supporting Information Additional supporting information can be found online in the Supporting Information section. Detailed sequencing quality control indicators for samples. [file IJOG-2026-4888573-s001.docx]

**Supplementary Table 1. Sample Information and Single-Cell RNA Sequencing Quality Control Metrics**

| **Sample ID** | **Group** | **Time Point** | **Total Cells** | **Median Genes per Cell** | **Median UMIs per Cell** | **Mitochondrial Reads (%)** |
| --- | --- | --- | --- | --- | --- | --- |
| S01 | Control | Healthy | 3,245 | 1,876 | 5,432 | 2.3 |
| S02 | Control | Healthy | 3,158 | 1,823 | 5,298 | 2.1 |
| S03 | MI | 7 dpi | 4,567 | 2,134 | 6,721 | 3.2 |
| S04 | MI | 7 dpi | 4,423 | 2,087 | 6,598 | 3.4 |
| S05 | MI | 14 dpi | 5,234 | 2,456 | 7,892 | 2.9 |
| S06 | MI | 14 dpi | 5,187 | 2,398 | 7,745 | 3.1 |
| S07 | MI | 30 dpi | 4,876 | 2,234 | 7,123 | 2.7 |
| S08 | MI | 30 dpi | 4,921 | 2,287 | 7,256 | 2.8 |
| S09 | ChrisKO | 7 dpi | 4,312 | 2,045 | 6,456 | 3.5 |
| S10 | ChrisKO | 7 dpi | 4,398 | 2,098 | 6,534 | 3.3 |
| S11 | ChrisKO | 14 dpi | 5,023 | 2,321 | 7,456 | 3.0 |
| S12 | ChrisKO | 14 dpi | 5,134 | 2,376 | 7,589 | 2.9 |

**Note:** *dpi = days post-infarction; UMI = Unique Molecular Identifier; MI = Myocardial Infarction; ChrisKO = gene knockout model. Quality control filtering was performed to retain cells with ≥200 genes detected and <10% mitochondrial reads. Total cells represent high-quality cells after filtering and quality control.*
